# A Bayesian Modelling Approach with Balancing Informative Prior for Analysing Imbalanced Data

**DOI:** 10.1371/journal.pone.0152700

**Published:** 2016-04-12

**Authors:** Kerenaftali Klein, Stefanie Hennig, Sanjoy Ketan Paul

**Affiliations:** 1 Clinical Trials and Biostatistics Unit, QIMR Berghofer Medical Research Institute, 300 Herston Road, Brisbane, Australia; 2 School of Pharmacy, University of Queensland, 20 Cornwall Street, Brisbane, Australia; University at Albany State University of New York, UNITED STATES

## Abstract

When a dataset is imbalanced, the prediction of the scarcely-sampled subpopulation can be over-influenced by the population contributing to the majority of the data. The aim of this study was to develop a Bayesian modelling approach with balancing informative prior so that the influence of imbalance to the overall prediction could be minimised. The new approach was developed in order to weigh the data in favour of the smaller subset(s). The method was assessed in terms of bias and precision in predicting model parameter estimates of simulated datasets. Moreover, the method was evaluated in predicting optimal dose levels of tobramycin for various age groups in a motivating example. The bias estimates using the balancing informative prior approach were smaller than those generated using the conventional approach which was without the consideration for the imbalance in the datasets. The precision estimates were also superior. The method was further evaluated in a motivating example of optimal dosage prediction of tobramycin. The resulting predictions also agreed well with what had been reported in the literature. The proposed Bayesian balancing informative prior approach has shown a real potential to adequately weigh the data in favour of smaller subset(s) of data to generate robust prediction models.

## Introduction

Well balanced datasets are very important to generate robust prediction models, especially in the fields of medical statistics. An imbalanced dataset is one of the main causes for the reduced generalisation of the relevant study findings. Conventional data analysis approaches do not take account of imbalance in a dataset. They weight the data points similarly when multiple subgroups of data are of individual interest. When a dataset is extremely imbalanced, the resulting prediction model will lose its robustness. The cost of sub-optimal prediction for smaller subsets is often higher than that of the bigger subset, which is especially true in medical datasets where high risk patients tend to be smaller subsets of data [1]. For example, Standing and his colleagues [2] highlighted that the analyses of adult PK data together with paediatric data to predict paediatric tobramycin PK might worsen the predictive performance. This may well be due to the imbalance in the corresponding dataset.

There are a number of techniques to address the imbalance issue [3]. The “under-sampling” is one of the proposed approaches, which samples the data from the dominant subset of the data by specific rules to ensure balance between the dominant and non-dominant groups[4]. While this approach has the potential to significantly reduce the computation time, there is a high likelihood of choosing a non-representative or biased subsample from the dominant group—reducing the generalisability of the inference. It is particularly true if the smaller subset of the data is indeed very small.

Similarly, another technique to tackle the imbalance issue is via “over-sampling”, where re-sampling approaches are used to increase the size of the smaller subgroup to achieve a balance with the larger subset of the data [5]. An advantage of this approach is the fact that all the data are used for analysis, however, it may require a very long computation time if the dominant subset of the data is substantially large.

In this study, we proposed a Bayesian alternative of “over-sampling”, via introducing a balancing informative prior in contexts of data characterisation and prediction. This was to weigh the data in favour of the smaller subset to equalise the influences of the dominant and smaller subset to the final prediction model, without lengthening the computation time. This new approach was evaluated via simulations in terms of bias and precision and further tested using a motivating example dataset in predicting the optimal dosage levels of tobramycin for various age groups.

## Methods

### Theory

In Bayes’ rule, the product of prior probability π(θ) and the likelihood of data given a parameter vector *f(y|θ)* result in the posterior distribution

$$\pi \left(\theta |\text{y}\right)\;=\;\frac{f\left(y|\theta \right)\;\pi \left(\theta \right)}{m\left(y\right)},$$

where *y* is the data and θ are the model parameters. The denominator *m(y)* is known as the marginal likelihood of the data and found by integrating the likelihood over prior densities

$$m\left(y\right)\;=\;{\int^{}}^{\text{​}}f(y|\theta )\pi \left(\theta \right)\;d\theta .$$

Depending on the dimensionality of θ and the complexity of *f*(·), the determination of the scaling factor *m(y)* is not often possible and the Markov Chain Monte Carlo (MCMC) [6] approach can be used in such cases. After a burn-in period which is necessary to converge from an initial parameter vector to the stationary distribution, each iteration of the MCMC approach represents a parameter vector out of the posterior distribution.

Suppose that the data *y* is characterised by a linear function

$$y_{i}\;=\;a+bx_{i}+\varepsilon_{i},$$

where the model parameters *a*, *b*, and the error term ε_i_ are assigned with appropriate non-informative prior distributions. Suppose that *x =* {*x*_*1*_, ⋯, *x*_*m*_, *x*_*m+1*_, ⋯, *x*_*n*_} where {*x*_*1*_, ⋯, *x*_*m*_} *<* {*x*_*m+1*_, ⋯, *x*_*n*_} and *m* << n, while the ranges covered by {*x*_*1*_, ⋯, *x*_*m*_} and {*x*_*m+1*_, ⋯, *x*_*n*_} are comparable. Then the conventional frequentist analysis of *y* will result in the inference which is dominated by {*x*_*m+1*_, ⋯, *x*_*n*_} and cannot be robust over the range of the smaller subset {*x*_*1*_, ⋯, *x*_*m*_}. Hence (*x*, *y*) is often divided into smaller subsets and analysed separately.

A Bayesian approach to address this common problem is proposed by introducing an auxiliary precision parameter *τ*_*int*,*i*_ *as* follows:

$$y_{i}\;=\;y_{int,\;i}+\varepsilon_{i},$$

$$y_{int,\;i}\;=\;a+bx_{i}+\;\gamma_{i},$$

where *γ*_*i*_*~N*(0, $\frac{1}{\sqrt{\tau_{int,\;i}}}$), {*τ*_*int*,*m*+1_, ⋯, *τ*_*int*,*n*_}are set to be a large constant φ, such as 10^6^, and {*τ*_*int*,1_, ⋯, *τ*_*int*,*m*_} are set to be φ • (*n—m*)/*m*. This *balancing* informative prior *τ*_*int*_ ensures that the influence of the larger subset on the inference being equal to that of the smaller subset. The default value of *τ*_*int*_ is set to be large so that the effect of introducing this otherwise unnecessary level of variability can be minimized.

### Validation of Theory

The validity of the introduced theory was evaluated through simulations and summarized via the bias and precision of posterior model parameter estimates. A dataset of 200 subjects’ body weights, aged from 2 to 25 years old were simulated using an Emax model [7] as follows:

$$Weight_{i}\;=\;A+B\;\times \frac{Age_{i}}{C+Age_{i}}+\varepsilon_{i},$$

where the Emax model parameters A, B, and C were set to be 2, 10, and 10 respectively. The Age vector was generated from a uniform distribution, ranging between 2 and 25 years old. *ε*_*i*_ was set to be normally distributed with mean zero and standard deviation of 0.25.

From the aforementioned dataset, 100 pairs of the small and the large subsets were simulated per a pre-defined fraction of the size of the small to the large subsets; the fraction was either 0.075, 0.1, 0.15, 0.2, 0.25, or 0.3. Each pair consisted of a smaller subset and a larger subset was created by first, selecting a set of (Weight_i_, Age_i_) where Age_i_ was less than or equal to 5 years old for the smaller subset and where Age_i_ was greater or equal to 18 years old for the larger subset. Then the size of the smaller subset was randomly reduced to each pre-defined fraction of the size of the larger subset, 100 times.

Finally, the model parameter values were re-estimated by defining

$$Weight_{i}\;=\;Weight_{int,i}+\varepsilon_{i},$$

$$Weight_{int,i}\;=\;A+B\;\times \frac{Age_{i}}{C+Age_{i}}+\gamma_{i},$$

where the prior distributions of A, B and C were set to be *N*(0, 1/$\sqrt{10^{6}}$), that of *ε*_*i*_ was N(0, 1/$\sqrt{\delta }$), *δ* ~ Gamma(0.001, 0.001), and *γ*_*i*_ ~ *N*(0, 1/$\sqrt{\tau_{int,\;i}}$). *τ*_*int*,*i*_ for Age_i_ greater or equal to 18 years old was set to be 10^6^ and that for Age_i_ less or equal to 5 years old was set to be 10^6^ • 1/the pre-defined fraction. The posterior distributions of the model parameters were obtained using the MCMC method.

The performance of the proposed method was evaluated in regards to bias and precision in the model parameter posterior estimates per 100 pairs of the small and the large subsets per each pre-defined fraction. The bias was calculated as the mean percentage of difference between posterior and true parameter values. The precision was calculated as the inverse variance of posterior model parameter estimates.

### Software/ Hardware used

For Bayesian analyses, Winbugs 1.4.3 [8] was used and for the simulations and the estimation of weight distribution across age, R 2.15.0 [9] was used.

### Motivating Example

The motivating example for this study was given by an imbalanced dataset with a larger number of data points obtained from adult patients and only a few data points from paediatric patients. The dataset used for this study contained the pharmacokinetic (PK) data after IV tobramycin administration to children and adults with and without cystic fibrosis (CF) which was previously analysed using a mixed effects modelling approach [10]. Hence, the following two compartment PK parameters [11]: individual clearance (CL), central volume of distribution (V1), distributional clearance (Q) and peripheral volume of distribution (V2) estimates for each patient were available. For the purpose of this study, these individual estimates were considered as data points to be analysed rather than the plasma concentrations in a typical PK analysis.

**Fig 1:**
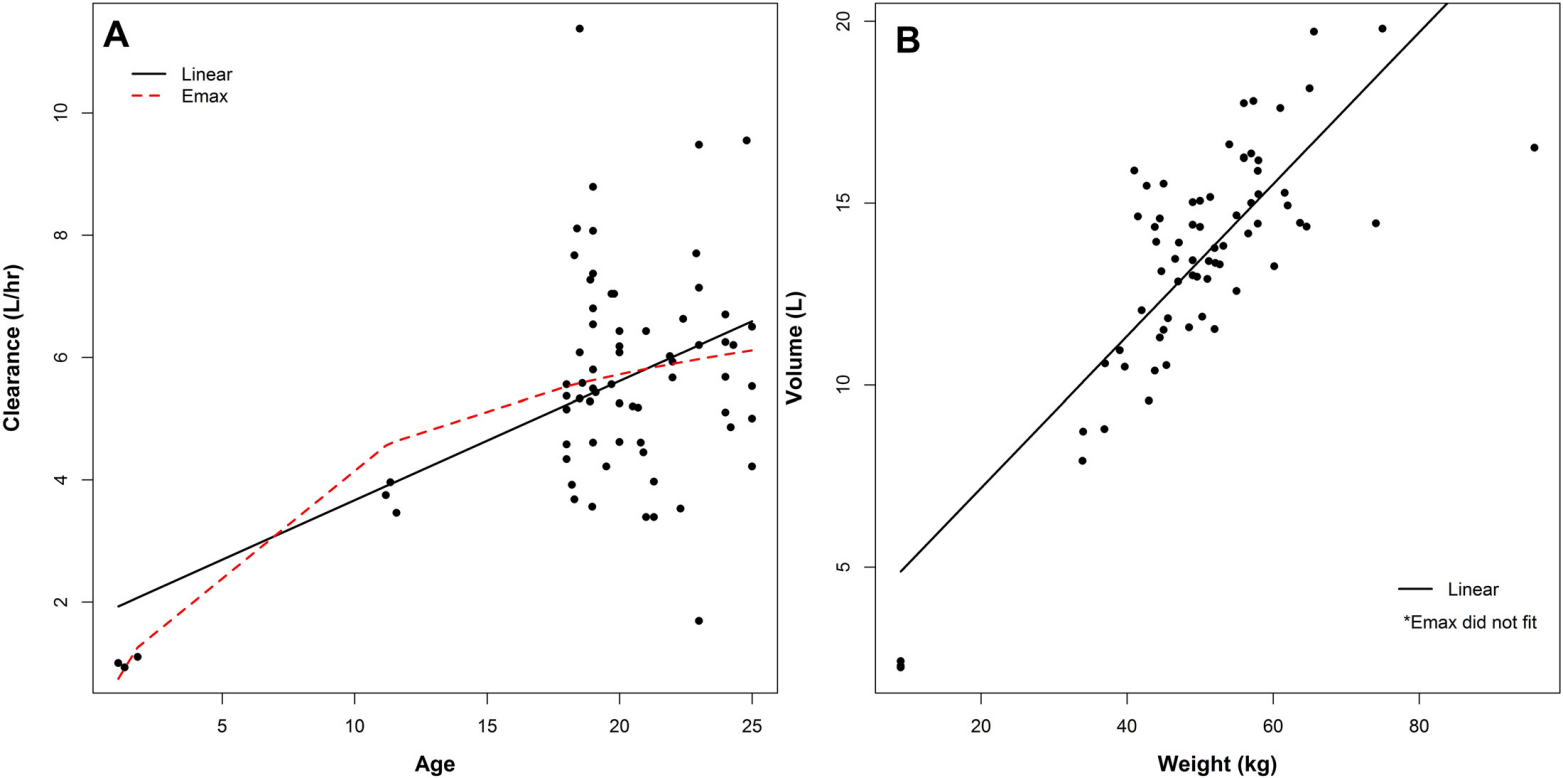
Linear and Emax model fits of individual tobramycin clearance (A) and volume (B) estimates from 63 adult and 6 paediatric cystic fibrosis patients.

From the whole dataset [10], only patients aged 1–25 years were included which resulted in data from 570 subjects (6.4 to 120 kg, 204 male patients) treated with tobramycin. From this dataset 100 subsets were randomly created for model building, each subset containing 63 adults (18–25 years), 3 children (10–13 years) and 3 infants (1–2 years). For example, black dots in Fig 1 are from one of the 100 model building subsets for CL (Fig 1A) and V1 (Fig 1B) versus age and weight respectively. Both conventional linear and Emax models were used to characterise the relationships between individual tobramycin CL versus age and V1 versus weight first.

#### PK parameter modelling

The central tendency of individual PK parameter estimates given a covariate was estimated using the Emax model

$$PK\;Parameter_{i}\;=\;PK\;Parameter_{int,i}+\;\varepsilon_{i},$$

$$PK\;Parameter_{int,i}\;=\;A+B\;\times \frac{Age_{i}\;or\;Weight_{i}}{C+Age_{i}\;or\;Weight_{i}}+\gamma_{i},$$

where V1, Q, V2 were modelled using weight and CL was modelled using age. The prior distributions of A, B and C were set to be *N*(0, 1/$\sqrt{10^{6}}$) and A is set to lie between 0 and infinity. That of *ε*_*i*_ was *N*(0, 1/$\sqrt{\delta }$), *δ* ~ Gamma(0.001, 0.001) and *γ*_*i*_ *~N*(0, 1/$\sqrt{\tau_{int,\;i}}$). *τ*_*int*,*i*_ for Age_i_ greater or equal to 18 years old was set to be 10^6^ and that for Age_i_ less or equal to 10 years old was set to be 10^6^ • 1/(0.1). This resulted in treating the data from the 6 paediatric patients of each model building subset as if from the 60 paediatric patients. Hence, with this balancing informative prior, the ratio of the numbers of paediatric and adult patients is roughly 1, instead of 0.1.

One hundred model building subsets of individual CL, V1, Q and V2 estimates from 6 randomly chosen paediatric and 63 adult CF patients were analysed using an Emax model as discussed earlier and the posterior model parameter estimates were generated from 10^5^ MCMC sampling after 10^5^ burn-in with thinning interval of 10.

The convergence of the Bayesian analysis for each model building subset was assessed by using the Geweke’s convergence diagnostic [12], Heidelberger and Welch’s convergence diagnostic [13,14], and graphical inspection of density and history plots of the posterior model parameter estimates. The appropriateness of this balancing informative prior approach was assessed using the residual sums of squares (RSS) and via graphical inspection.

#### Plasma concentration simulation

Once the posterior estimates of *A*, *B* and *C* were obtained for each model building subset, 10^4^ posterior predictions for CL, V1, Q and V2 at each age from 1 to 25 years were generated using the following principle.

$$PK{\overset{⌢}{Parameter}}_{i}=\;A+B\;\times \frac{Age_{i}\;or\;Weight_{i}}{C+Age_{i}\;or\;Weight_{i}}+\;\omega_{i},$$

Where *A* ~ *N*(*A*_*post*_, *ϕ*_*A*.*post*_), *B* ~ *N*(*B*_*post*_, *ϕ*_*B*.*post*_), *C* ~ *N*(*C*_*post*_, *ϕ*_*C*.*post*_) and *A*_*post*_, *B*_*post*_, *C*_*post*_ were the corresponding posterior mean estimates and *ϕ*_*A*.*post*,_ *ϕ*_*B*.*post*,_ *ϕ*_*C*.*post*_ are the posterior standard error estimates of the model parameters respectively. *ω*_*i*_ ~ *N*(0, *η*) where *η* is the observed inter-individual standard deviation of each PK parameter from the adult data only.

In order to generate 10^4^ posterior predictions of V1, Q and V2 per age group, body weight distribution across the range of age should be established first. The central tendency of weight distribution across age, with increment by 1 year, was estimated from the whole data using lowess smoothing function [15]. The inter-individual standard deviation of weight was obtained from 60 infant patients aged 1 to 2 years from the whole data.

Once 10^4^ posterior predictions of all four PK parameters per age were available from the model building subset, plasma concentrations at 1 hour and at 24 hour post single intravenous infusion (0.5 hours) of various tobramycin dose levels were predicted using a standard two compartment PK model [7].

#### Optimal dosage recommendation

An optimal dose was defined as the intravenous tobramycin infusion dosage (infusion duration of 0.5 hours) that provided the largest amount of patients achieving the following targets: a 1 hour post-dose plasma concentration of tobramycin which is 10 times higher than the minimum inhibitory concentration (MIC) and the 24 hour post-dose plasma concentration equals to or is less than 1 mg/L.

Plasma concentration time profiles of 10^4^ CF patients per age group were simulated using the analyses results from the 100 randomly generated model building subsets and optimal dosages were calculated for the selected age groups.

## Results

### Simulation

The estimations using balancing informative prior performed significantly better than the conventional approach in terms of bias and precision (Table 1). It is noteworthy to mention that the precisions of parameter estimates via the conventional approach is noticeably smaller compared to those from the balancing informative prior approach, suggesting that the utility of the resulting models from the conventional approach for generating robust predictions would be limited. Moreover, the proposed methodology worked as a Bayesian generalisation of over-sampling with a clear advantage of not requiring extra computation time.

**Table 1:** Simulation results showing bias and precision of three estimated model parameters (A, B, C) from the Emax model. The ratio of the smaller subset size to the larger subset size is represented in fractions.

Modelling with Balancing Informative Prior						
Fraction	**Bias (%): A**	**Bias (%): B**	**Bias (%): C**	**Precision: A**	**Precision: B**	**Precision: C**
**0.075**	0.22	-0.12	-0.43	313137.2	78074.9	37888.1
**0.1**	0.33	-0.36	-0.25	150363.3	47769.29	49410.27
**0.15**	-0.30	0.10	-0.34	136159	37351.8	24623.67
**0.2**	-1.02	0.36	-0.27	143532	44390.92	43733.94
**0.25**	0.05	-0.17	-0.06	159675.2	47285.89	46163.66
**0.3**	0.28	-0.08	0.59	135889.7	39767.15	23996.88
Conventional Modelling						
Fraction	**Bias (%): A**	**Bias (%): B**	**Bias (%): C**	**Precision: A**	**Precision: B**	**Precision: C**
**0.075**	-6.33	-0.81	-5.45	4.48	17.96	0.33
**0.1**	-16.35	-0.60	-12.98	2.36	7.88	0.31
**0.15**	-20.07	-0.78	-17.09	4.04	21.59	0.40
**0.2**	-10.02	-0.99	-9.52	6.64	53.30	0.43
**0.25**	-7.70	-1.08	-7.82	7.27	68.77	0.47
**0.3**	-3.14	-1.32	-4.91	12.73	99.45	0.68

### Motivating example

Fig 1 showed that using the conventional model fitting approaches, the estimated central tendency of the sparse paediatric data across age related covariates was influenced by the rich adult data. The clearance in children was overestimated by the Emax model and the linear model and V1 for infants was overestimated by the linear model, essentially treating sparse paediatric data points as outliers.

**Fig 2:**
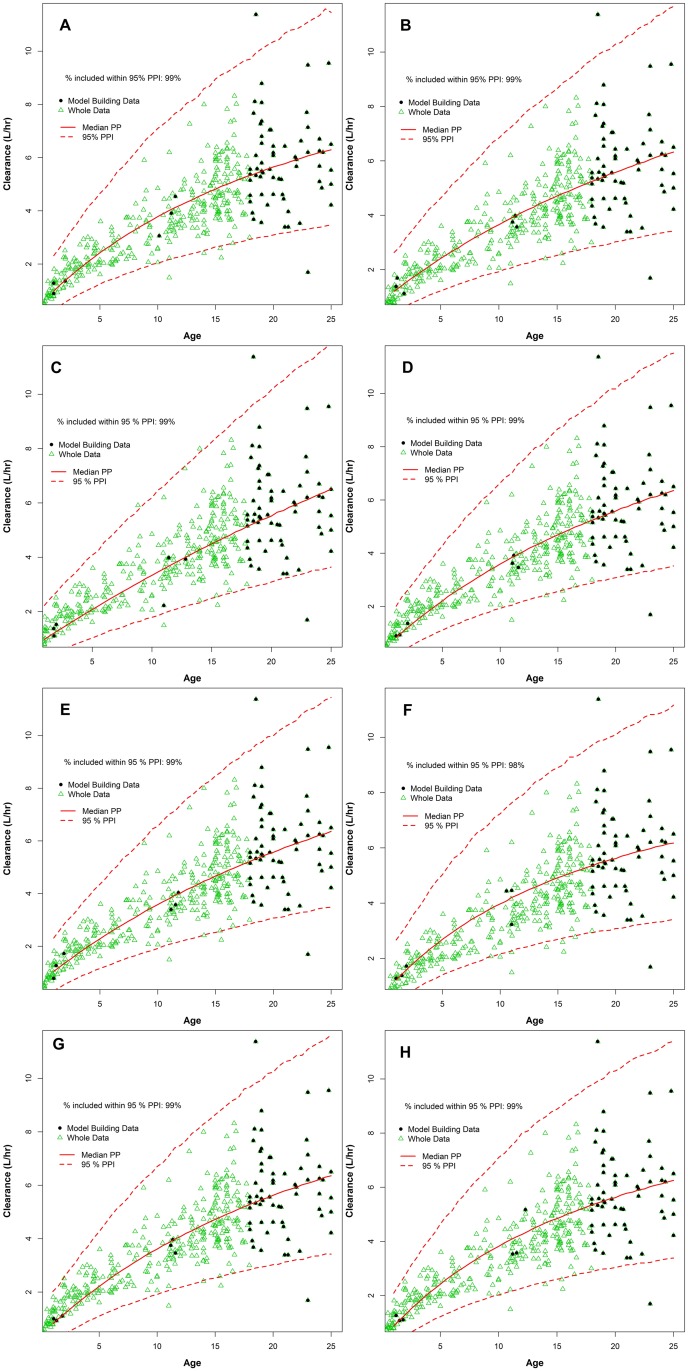
Median and 95% posterior prediction intervals of tobramycin clearance versus age based on Emax model fits for eight model building subsets of 63 adult and 6 randomly chosen paediatric cystic fibrosis patients respectively.

Fig 2 presented the prediction of individual CL estimates based on the proposed Emax model for the first eight of 100 model building subsets along with the surrogate inter-individual standard deviation estimate from the adults only data, compared to the whole data. The convergences of the fits were achieved in 90 of the 100 sets of model building data. The proposed Bayesian modelling approach with the balancing informative prior described observed limited paediatric data well and the 95% posterior prediction intervals covered the whole data. The 95% posterior prediction intervals covered 98 to 99% of the whole data in all eight cases.

**Fig 3:**
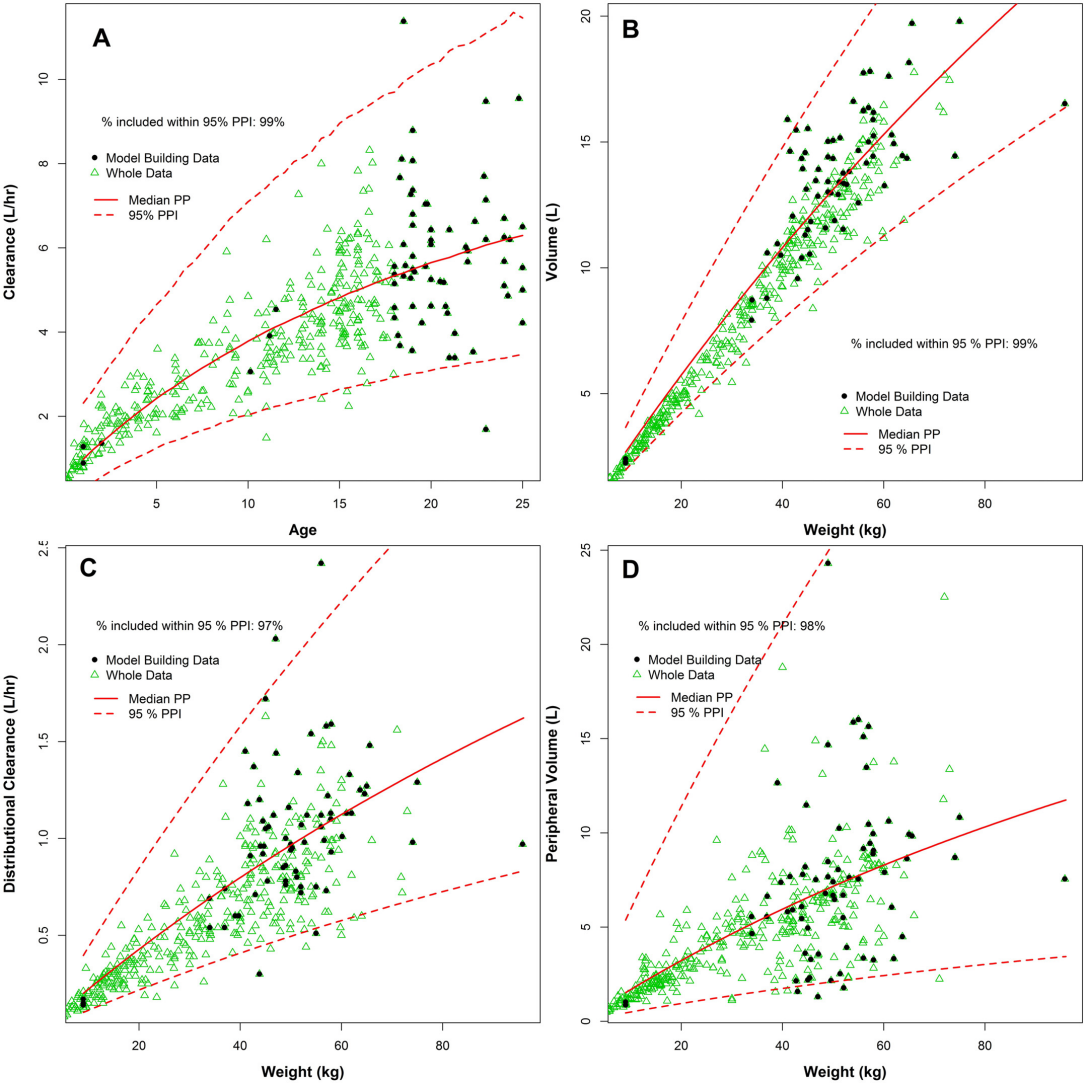
Median and 95% posterior prediction intervals of tobramycin clearance (A), volume (B), distributional clearance (C), and peripheral volume (D) versus age/weight based on Emax model fits for a model building subset of 63 adult and 6 randomly chosen paediatric cystic fibrosis patients, respectively.

Fig 3 presented the 95% posterior prediction intervals for all four PK parameters estimates based on the analyses of a model building subset, included data from 69 patients, compared to the whole data (570 patients). Again the coverage of the intervals was very good (97–99% coverage), demonstrating a potential for the proposed approach to be used to build a conservative population PK model.

Plasma concentration time profiles of 10^4^ CF patients per age group were simulated using the analyses results from each of the 100 randomly generated model building subsets and optimal dosages were calculated for the selected age groups (Table 2; eight sets of predictions only). Minimum effective tobramycin dose recommendations with minimum 90% optimal dose criteria acceptance rate lied mostly between 11 and 12 mg/kg/day (84%) which agreed well with the optimal dosage recommendation by Hennig *et al*. [10] and by others with different optimal dose criteria [16,17]. If we considered the minimum dose recommended across age groups per each model building subset, the doses that would be recommended to the paediatric patients were 10–11 mg/kg/day, based on 100 model building subset analysis results (Table 2; eight sets of predictions only).

**Table 2:** Minimum effective tobramycin dose recommendations (mg/kg/day) with minimum 90% optimal dose criteria acceptance rate based on 10,000 CF patient simulations, using the analysis results of the first eight model building subsets (MBD).

	Minimum Effective Dose (mg/kg/day)							
Age	MBD 1	MBD 2	MBD 3	MBD 4	MBD 5	MBD 6	MBD 7	MBD 8
**1**	12	12	11	11	12	12	12	12
**2**	12	12	11	11	12	13	11	12
**3**	12	12	11	11	12	13	11	12
**6**	12	12	11	11	11	12	11	12
**9**	11	11	11	11	11	12	11	12
**12**	11	11	11	11	11	11	11	11
**15**	11	11	11	11	11	11	11	11
**18**	11	10	11	11	10	11	11	11
**21**	11	11	11	11	11	11	11	11
**25**	11	11	11	11	11	11	11	11

## Discussion

In this study, we demonstrated that (1) the conventional approach to analyse imbalanced datasets is inadequate, and (2) the proposed Bayesian balancing informative prior approach could be viewed as a Bayesian generalisation of over-sampling approach to weigh the data in favour of minor under-represented subsets. We have established the potential utilities of the proposed Bayesian approach through extensive simulations.

**Fig 4:**
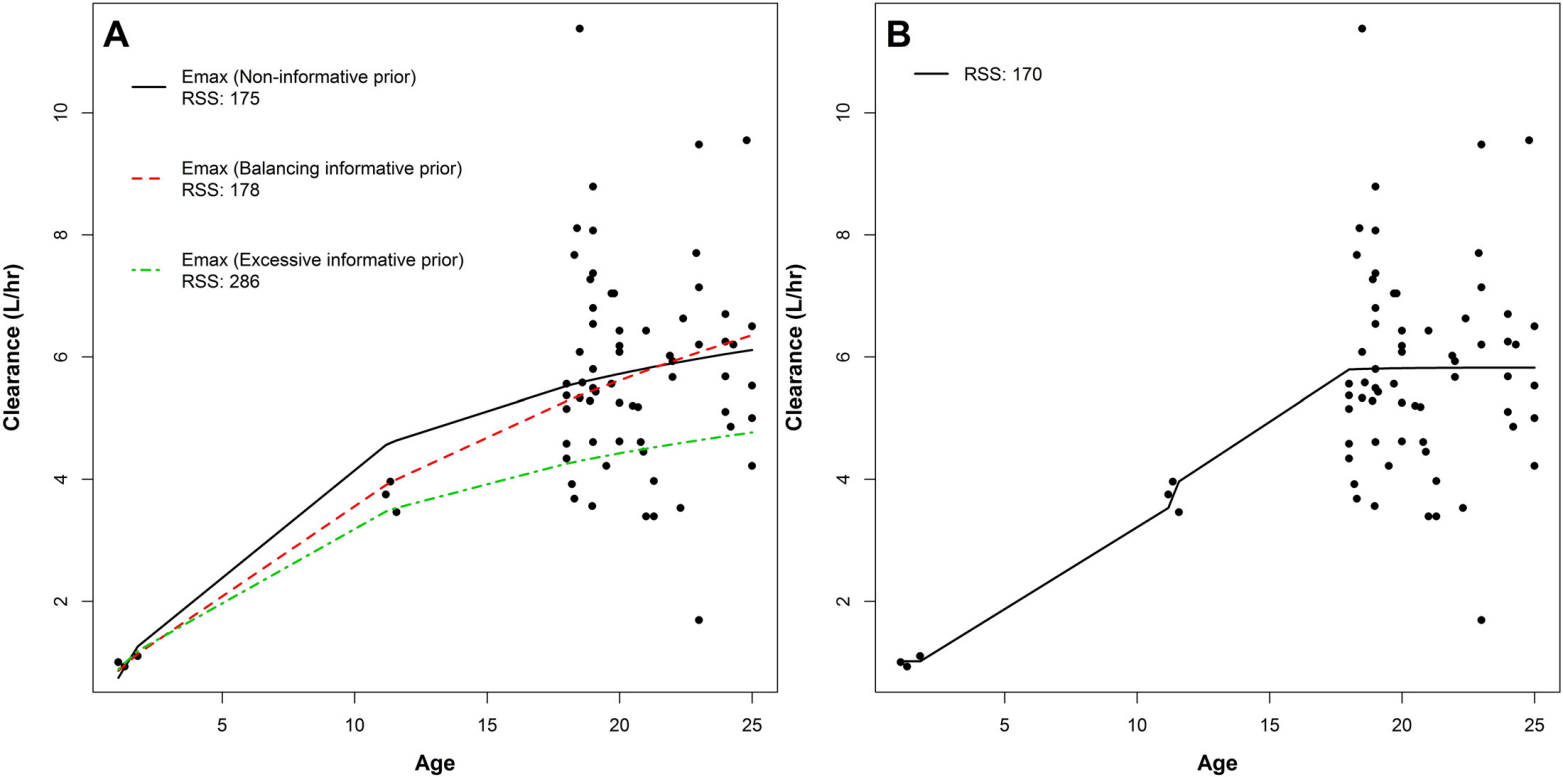
Emax model fits using various degrees of informative priors on the paediatric data points (A), and a sigmoidal Emax model fit (B) of individual tobramycin clearance estimates versus age from 63 adult and 6 paediatric cystic fibrosis patients. RSS stands for residual sums of squares.

It can be argued that the best information on the minor subset should come from this population predominantly, and not be over-influenced by the dominant subset of the data. Using the context of motivating example, Fig 4A demonstrated that the balancing informative prior approach adequately characterise the central tendency of paediatric CL estimates over the age without being over influenced by the adults’ data. Using the balancing informative prior, it was also shown that the paediatric data was not over-influencing the fit of adult population as the excessive informative prior (*τ*_*int*,*i*_ for the paediatric patients: 100 fold of *τ*_*int*,*i*_ for adults) would. Residual sums of squares (RSS) produced using the excessive informative prior showed that the corresponding fit did not adequately characterise the adult data any more (Fig 4A).

In the motivating example, only six paediatric patients, 3 children and 3 infants, were included in the model building subset as a minimum amount of paediatric data necessary to apply this methodology. It is advised not to analyse only adult and sparse infant data as the corresponding estimated central tendency for children’s PK parameters would be largely dominated by a functional form of a model used (e.g. Emax). However, the method is still applicable if one has only rich adult and sparse adolescent/children data. Minimum six paediatric data points were worked out by considering crude probability that all 6 data points were greater than the 90% confidence bound of a parameter being 1.5 E-8 (i.e. 0.05^6^), which is a very unlikely event. It is also noteworthy that this study was done to improve methods for interpolation between sparse paediatric data and rich well known adults’ information only.

The Emax model was chosen as a structural form to fit all four two compartment PK parameters against age related covariates, which is an empirical model. Emax model was assumed to be a flexible model to adequately characterise the central tendency of PK parameters across an age related covariate, given that the expected tendency was a monotone increase or decrease over the studied range of the covariate. This could be relatively easily assumed through adequate physiologically-based PK modelling. Use of sigmoidal Emax models [11], which has been suggested as mechanistic models for extrapolation spanning neonates to adults [18], are not recommended for the interpolation with sparse paediatric data. This was clearly demonstrated in Fig 4B.

Although we were using very limited paediatric data, the convergences of the fits were achieved in 90 of the 100 sets of model building data. Four out of the first eight model building subsets analysed had minor convergence issues. However, these issues did not result in lack of posterior predictive interval coverage for the whole data as demonstrated in Fig 2, which was the ultimate objective of a model fit in this report.

In relation to optimal dosing, a MIC value of 2 mg/L was set for this study based on the European Committee on Antimicrobial Susceptibility Testing database reporting 91.9% of pseudomonas aeruginosa showing a MIC of ≤ 2 mg/L for tobramycin [19]. It was previously suggested that the most predictive PK/pharmacodynamic relationship of clinical outcome for aminoglycosides [20] and for tobramycin in CF patients is the maximum concentration/MIC [20,21]. Others have shown that achieving an aminoglycoside free-drug maximum concentration/MIC ratio of ≥ 10 within 48 hours of initiation of therapy for gram-negative pneumonia resulted in a 90% probability of therapeutic response by day 7 [22]. Additionally, it has been shown that low trough concentrations are associated with a lower potential risk of toxicity [23].

This report proposed an approach to analyse imbalanced data using Bayesian balancing informative prior approach. Our finding suggested that even when there is a severe imbalance in number of subjects available from different covariate sub-ranges, a joint analysis is feasible and a robust conclusion can still be drawn.
